# Biology, geometry and information

**DOI:** 10.1007/s12064-021-00351-9

**Published:** 2021-06-11

**Authors:** Jürgen Jost

**Affiliations:** 1 Max Planck Institute for Mathematics in the Sciences, Leipzig, Germany; 2 Santa Fe Institute for the Sciences of Complexity, Santa Fe, New Mexico USA

**Keywords:** Control, Regulation, Biological information, Three-dimensional geometry, Principle of life, Biological process, Complexity

## Abstract

The main thesis developed in this article is that the key feature of biological life is the *a biological process can control and regulate other processes*, and it maintains that ability over time. This control can happen hierarchically and/or reciprocally, and it takes place in three-dimensional space. This implies that the information that a biological process has to utilize is only about the control, but not about the content of those processes. Those other processes can be vastly more complex that the controlling process itself, and in fact necessarily so. In particular, each biological process draws upon the complexity of its environment.

## Introduction

This is an essay about the conceptual foundations of modern biology and the role that mathematics can play for biology.

Traditionally, two aspects have been considered as fundamental for or constitutive of life, reproduction and metabolism. The concept of evolution puts the emphasis on the first of them, reproduction. Some modern versions, like the notion of the selfish gene, go well with the general public, but fall short of capturing the complexity of life. An important property of biological reproduction is the transmission of information, rather than of material structures. Other approaches, like autopoiesis or dynamics far from thermodynamical equilibrium, put more emphasis on the second aspect, metabolism, that is, maintaining a biological organism and preventing it from disintegrating. Metabolism needs a constant inflow of matter and energy, not just of information.

In this article, I wish to develop a conceptualization that combines and intertwines the two aspects. I shall propose that the key feature of biological life is the control and regulation of processes. This can happen in a hierarchical or a reciprocal manner. The basic processes themselves are material and occur in time and space, three-dimensional space in fact. The latter will assign a more fundamental role to geometry than usually allowed for in theoretical biology. The control and regulation of processes, while possibly depending on material interventions, requires information, about which processes to select and how to control them, so as to satisfy the needs of the controller to build up and maintain its structure. Importantly, the controller thereby externalizes much of its requirements and makes itself dependent on complex other processes in its environment. Complex life can only survive in a complex environment. In many regards, that environment has to be more complex than the controller itself. An extreme biological example are viruses that are entities consisting of a simple mechanism to control possibly very complex organisms for the purpose of their own proliferation. But control can also simply consist in the utilization of basic physical laws, like gravity, or properties of three-dimensional space. The general principle is that what can be provided for by the physical, chemical, biological or perhaps social environment need not be manufactured by the system itself. Since this principle can be iterated in the biological and social realm, ever more complex structures can build up in a hierarchical manner or may depend on each other in a reciprocal manner. Externalization by substituting the shaping and exploitation of external processes for internal ones, and internalization by the tighter control of originally independent processes then go hand in hand, as also emphasized in Laubichler and Renn (2015). In fact, in Laubichler and Renn (2015) this is described as the interplay between niche construction and regulatory networks.

This paper is an extended version of my presentation at the Conference “Geometry and Phenomenology of the Living. Limits and possibilities of mathematization, complexity and individuation”. I thank Luciano Boi, Carlos Lobo and Giuseppe Longo for organizing a very stimulating meeting. I also thank Klaus Scherrer for an inspiring collaboration on the conceptual foundations of molecular biology, and Manfred Laubichler and many others for stimulating discussions.

## Biology and mathematics

Geometry, information, dynamics, etc., that is, fundamental concepts that emerged in the introduction, are mathematical concepts. We shall consequently systematically draw upon mathematical thinking. To put this into perspective, let us first discuss the general question of what mathematics can contribute to biology. Some general possibilities are

- Methods for detecting structure in data
- Dynamical models of biophysical processes and their analysis
- Abstract conceptual analysis

Obviously, these approaches operate at very different levels. Therefore, let us consider some possible mathematical approaches in more detailed terms. We shall then see that in some sense, they cut across the different levels of the preceding list.

1. Information theory
2. (Three-dimensional) geometry
3. Biophysical models and dynamical systems
4. Network analysis and generalizations, like simplicial complexes or hypergraphs

Let us discuss them in some more detail.

1. Information theory: Here the biologically fundamental question becomes what information is relevant. That is, information acquires its value only insofar it is related to the biological entity in question, by guiding its survival, maintenance or reproduction.
2. (Three-dimensional) geometry: The fact that life occurs in three-dimensional space is important, but often not addressed at all in theoretical biology. It is, however, clearly expressed and explored in Bailly and Longo (2011). 3D enables, facilitates and/or constrains biological processes. Fewer dimensions would offer too few possibilities for spatial arrangement or interaction, while more dimensions might not constrain biological processes enough to prevent them from disintegrating.
3. Biophysical models and dynamical systems: Here, an important question is about the appropriate level of detail of the biological models. In fact, more detailed models sometimes yield less accurate or robust predictions than coarser ones. Some of this may simply be cases of overfitting, but the deeper reasons are not yet systematically understood from a biological perspective.
4. Network analysis and generalizations, like simplicial complexes or hypergraphs: Here, an important point that is quite generally ignored in the analysis of biological and other networks is that the edges express relations, and they, in place of vertices, should therefore be the basic objects of network analysis. In particular, the quantities utilized in network analysis should assign values to edges, rather than to vertices.

## Three-dimensional geometry

Biological structure exists and interacts in space. Space is 3D, although from the perspective of biological organisms not Euclidean, because gravity acts in one direction on the surface of the earth, and therefore, the degrees of mobility of and interactions between terrestrial organisms are often constrained to something more like 2D. On the scale of cells, this plays a more minor role. Nevertheless, one of the most important structures, the DNA is arranged in a one-dimensional manner. Why is this so? It is not 3D, because the interior of a 3D object is not accessible for readouts or copying. It is not 2D, because a linear structure is better adapted to sequential, temporal processing. That in turn is needed because there are bottlenecks like the ribosome where polypeptides are assembled. Likewise, replication seems to be less error prone and more energy efficient when arranged in a sequential, temporal manner instead of taking place simultaneously, like in a Xerox machine. When replication is carried out sequentially, the same copying molecules and structures can be used repeatedly. Of course, both the DNA itself and also its products, the polypeptides then acquire a 3D shape. For the polypeptides that constitute proteins, this is essential for their biological functions, because in that way particular motives can be exposed to interactions with other substances or shielded from such interactions. For the DNA, the spatial arrangement is important for the regulation of gene transcription, as emphasized by Boi (2011). For instance, via a suitable spatial organization, genomic regions that should be simultaneously transcribed can be brought into spatial proximity, even if their intrinsic linear distance on the DNA could be quite large, thereby facilitating coregulation, as originally proposed by Képès and Vaillant (2003), although their original solenoid model may have been too simple. The spatial organization of the DNA is certainly not as erratic as it originally looked, nor as regular as proposed in the first models, but very carefully orchestrated by specific proteins.

For the RNA, which is not only an intermediate between the DNA and proteins, but the crucial instance for regulation and processing (as well as for a host of catalytic roles), the secondary structure is important, achieved by pairwise bonds between complementary nucleotides in a linear sequence. Much of the processing is regulated by interactions with specific proteins, and in Jost and Scherrer (2014), a combinatorial code has been proposed. In turn, RNA molecules can also function as scaffolds for bringing specific groups of proteins together to induce their functional interaction. For that role, a 2D structure seems to be the most appropriate.

A caricature might then say that we proceed from the 1D DNA (information storage) via the 2D RNA (regulation and processing) to the 3D proteome (cellular function). Of course, as discussed, the DNA is organized in 3D, and RNAs and proteins not only have a 3D shape, but also interact in 3D.

The latter point indicates that 3D geometry is important not only for single structures, like proteins or the DNA, but also for the interaction between structures. It facilitates and constrains interactions at the same time. In 3D, substances can find each other more easily than in higher dimensions, but there are also constraints for simultaneous interactions (see Bailly and Longo 2011, p.122, on this point). If some substance occupies a place in space, that place is no longer accessible to others. The effects may not always be easy to access. Let us consider the example of the toponome project of Walter Schubert, Andreas Dress and their collaborators (Schubert et al. 2006). By repeated staining and bleaching of a cell slice, they can record the positions of about 100 proteins in that slice. In particular, one then has data about the colocalization of proteins. These data can be arranged in a simplicial complex. The vertices of that simplicial complex stand for the various proteins. Two vertices are connected by an edge if the two corresponding proteins frequently occur in neighboring positions. Here, one can set some threshold, how often those proteins should occur together in order to speak of cooccurrence and introduce the corresponding edge. Similarly, one inserts a two-dimensional simplex, that is, a triangle with three vertices, when all three corresponding proteins frequently occur together, and not only each pair among them. And similarly for higher-dimensional simplices. Proteins can interact only when they are in spatial proximity, that is, when they cooccur, and so, this simplicial complex represents some kind of geometric backbone for the interaction patterns. Of course, interactions are realized by chemical affinities. This in turn leads to the question which of those potential chemical reactions are actually realized in the cell. 3D geometry may prevent certain chemically possible interactions from happening, because not all of them can happen simultaneously in space. The mathematical question then is what constraints this creates for the topology of the simplicial complex whose construction we have just described. To study such a simplicial complex, Betti numbers (dimensions of homology groups in algebraic topology, see for instance Jost (2015), [14]) and geometric invariants like Laplacian spectra (Horak and Jost 2013) can be used for qualitative comparison of colocalization patterns in different cells (e.g., healthy vs. diseased).

At another scale, the organization of the brain is also three-dimensional. Since not every structure can be in spatial proximity with every other structures, more distant structures need to be connected by biological wires or cables. Sending information through such cables takes time, and this then slows down the processing speed for signals entering the brain. Making the cables thicker increases the speed, but then fewer such cables can fit into some given region. Therefore, there is an optimization problem for the arrangement of the various cortical and subcortical structures and the wiring between them, so that the most important signals can be processed as fast as possible. But since those important signals and the adequate responses to them may be quite heterogeneous, compromises between the processing efficiencies of various data are necessary. Shaped by different structural constraints and channeled by historical contingencies, different brain architectures have evolved, from the distributed brains of cephalopods to the intricately folded neocortex of mammals that sits on top of and interacts with evolutionarily much older structures like the cerebellum, the basal ganglia or the hippocampus. The avian brain is much smaller and, importantly, lighter than the mammalian one and structurally differently organized, but capable of comparable intelligence. We may then ask how good the solutions are that biological evolution has found for the spatial organization of the brain, or whether another, perhaps radically different or more systematic design might be superior for the problems that the brains of current organisms have to handle.

A closed surface can shield its interior from external perturbations or influences. This inaccessibility has positive and negative aspects. An obvious positive aspect, emphasized for instance in Maturana’s and Varela’s theory of autopoiesis, is that a cell wall prevents the cell from disintegrating and at the same time, being selectively permeable enables the inflow of needed material. But then also interactions with external substances that should not or cannot be admitted into the cell need to be mediated by receptors on the cell wall and internal signaling cascades.

And we also recall that inside the cell, the DNA could not be intrinsically three-dimensional, as otherwise it would not be accessible for transcription and replication.

We conclude that information, regulation and geometric structure are interwoven, and each theoretical treatment should keep that in mind.

## Interactions, networks and hypergraphs

Colocalization patterns of proteins constitute temporal snapshots. They constitute preconditions or show the results of metabolic or other biochemical reactions. In those reactions, also other substances are involved, and the proteins may catalyze those reactions. These reaction sets are properly modeled not as simplicial complexes, but as *chemical hypergraphs*. These are structures where two sets of vertices, standing for educts (ingredients) and products of chemical reactions, are connected by hyperedges, standing for the chemical reactions. These sets of vertices need not be disjoint, as catalysts should be counted as both educts and products of reactions. The formal analysis of such chemical hypergraphs has been started in Jost and Mulas (2019). Such hypergraphs can be analyzed via Laplacian spectra or by the distribution of metric curvatures. Chemical reaction networks are constrained by stoichiometry. In this regard, a theory has been developed by St.Schuster and others for decomposing metabolic pathways into elementary modes, see for instance (Klamt and Stelling 2003; Schilling et al. 1999; Schuster et al. 2000; Schuster and Hilgetag 1994). The availability of external ingredients and energy (provided by ATP) and reaction rates, but also spatial organization, constrain how much can be produced in parallel or sequentially. Again, coordination, regulation, and control are necessary.

## Regulation

As we have already seen, the coordination of processes can be achieved in principle by spatial proximity (geometry), or by joint signals (information). These are not alternatives, but can be flexibly combined.

And spatial interactions may have a dual role. We again recall the example of the interaction of RNAs and proteins (RNPs). There are two possible functional roles:

1. The RNA serves as a scaffold for protein interactions. Thus, a specific spatial organization of the cell can guide the specificity of interactions, or
2. the regulation of gene expressions via a combinatorial code, for the coordination of expressions of specific collections of genes.

The first item emphasizes again the role of topology. Klaus Scherrer and myself have therefore proposed the term *topon* for a geometric configuration of regulatory significance. The second item is systematically developed in Scherrer and Jost (2007a, b, 2009). An important biological principle is that (pre-)mRNA is only further processed when some binding proteins are removed. That is, the removal of individual proteins shared by a specific collection of mRNAs enables the coordinated activation of specific sets of genes. Here, we see the power of combinatorics (Jost and Scherrer 2014). The binding motifs for those proteins are contained in the RNA sequence, and so, one and the same stretch of RNA may have both a coding and a regulatory role, and we have proposed the term *genon* for such a regulatory motif superimposed on a coding sequence. An mRNA has about 20 such binding sites for proteins, each of them shared with some other RNAs. Thus, taking for instance 5 binding sites, there is a specific group of mRNAs that have all of them in common. When all binding sites are occupied by their corresponding proteins, the mRNA is not further processed, but sits there in some kind of dormant state. When, however, a certain number, say 5, of those proteins is removed, the mRNA is further processed and translated into a polypeptide. Thus, when some signal removes those 5 proteins from all their binding sites, a specific group of mRNAs is translated. In other words, we have some combinatorial scheme that enables the cell to translate a specific set of genes, according to specific requirements. The numbers involved, of different binding sites, of binding sites per mRNA and of proteins to be removed for processing, are such that there is a huge number of combinatorial possibilities. See (Jost and Scherrer 2014) or (Jost 2014) for details.

The principle behind this can be formulated more abstractly, as the suppression of inhibition. The natural tendency of DNA is to be transcribed into RNA, and in turn that of RNA is to be translated into a protein, that of a protein to execute its function and that of a cell to proliferate. But if all RNAs in a cell are translated, all proteins are active, and all cells in a tissue or an organism proliferate, total chaos will result, and the cell or the organism will become dysfunctional. In any given context, only a small, but specific fraction should be active or proliferate. Therefore, as a general rule, activity should be inhibited, and only when the situation requires it, that inhibition should be suppressed. Thus, most of the DNA in a cell is not available for transcription, but shielded by heterochromatin. As described, translation of RNA is inhibited by proteins or other, non-coding RNAs. For proteins, we have allosteric inhibition (Monod et al. 1963) where another protein binds to a non-functional region of a protein and thereby inhibits its activity, and before the protein can become active, that other protein needs to be removed, usually by still another protein. The uncontrolled proliferation of cells in an organism is cancer, and as Longo (2018) argues, for understanding cancer, the paradigm that everything is steered by the DNA, and here specifically, that DNA mutations cause uncontrolled cell proliferation, is inadequate, and one should rather understand what regulation mechanisms suppress the inhibition for cell division. Similar ideas are widely discussed within so-called evolutionary medicine. Also, in social animals, typically reproduction of group members is inhibited, and only some very few selected individuals are allowed to produce offspring, although here I do not want to go into the controversial issue of group selection.

## A fundamental thesis

We shall now formulate our fundamental thesis (see also Jost 2019a, b for different contexts) and explore its consequences.

### Thesis 1

*The key principle of biology is that a process can control and regulate other processes.*

**Examples:**

- Promoter, repressor, etc., sites at the DNA are unspecific for the coding regions, but reflect the regulation schemes
- There are many general combinatorial regulatory mechanisms at RNA level (interactions between different RNAs or RNAs and proteins), some of which are described in Sect. Regulation
- Hoxgenes as general regulatory mechanisms across species (Gehring 1998)
- Principle of allosteric inhibition (Monod et al. 1963) as discussed in Sect. Regulation
- Insects have a general, unspecific control mechanism for transforming sensory input in motor activity. They can therefore flexibly couple sensors to actuators.
- In the research direction of Evo-Devo (which can be seen as a challenge to the Neodarwinian paradigm), the key is the reorganization of control mechanisms (see for instance Carroll et al. 2005; Laubichler 2007)

These examples suggest our next thesis.

### Thesis 2

*The content of these controlled processes matters only insofar as it serves the controlling process.*

This then has implication for the question “*What is relevant information?*”. It leads to a new concept of biological information.

### Thesis 3

*Relevant information is only what is needed for regulation and control. This may be very little. But the system needs to be capable of storing, memorizing or preserving that information.*

Let us discuss some examples and applications.

1. A virus, in order to start with perhaps the most extreme example, only needs to “know” how to find a host and inject its DNA or RNA into that hosts cells. Therefore, the genetic information of the virus can be very short. The virus controls the host’s metabolic processes to ensure its own replication. How those processes operate is irrelevant.
2. A higher animal, a mammal for instance, has the evolutionary choice about which metabolic products to manufacture itself and which to simply take in as food. Vitamins are a good example. They are essential for the metabolism, but their production is externalized. Thus, the animal no longer needs to store the information about the necessary metabolic processes in its genome, but rather the information how to acquire food containing the necessary vitamins.
3. A common aspect of the two preceding examples is that a biological organism or process (if we may consider the replication of a virus as a biological process) depends on an environment that may be vastly more complex than itself. The metabolic information about how to produce vitamins may be much higher than the information about finding the appropriate food source, but only the latter is needed for the organism or process.
4. Biological organisms not only exploit other organisms or processes in their environment, but also, and perhaps even more basically, physical laws and regularities. For instance, gravity is actively exploited in much of animal locomotion. Our bodies are adapted for walking in the presence of gravity of a very particular strength. Robotics has recently learned to also utilize the forces of gravity, instead of carefully programming the positions of all the joints of a walking robot. That is called *embodiment*.
5. As a consequence of the principle that a biological organism depends on both a complex environment and on the operation of physical laws, it is doubtful whether we can ever establish human life on other planets. While we may be able to control other physical parameters like the temperature or the oxygen supply, our bodies are not adapted to operate under a different gravity strength. And whatever artificial biological environment we may be able to create, it may not be complex enough to sustain human life in the long term.
6. Ashby’s law of requisite variety (Ashby 1956) is incorrect. That law says that a system needs to maintain enough variety to match all external perturbations if it persists in the presence of such perturbations. In fact, as a consequence of our theses, the system needs much less. It simply needs to control processes, either directly those that generate the perturbations, or others that handle those perturbations.
7. Most of the preceding examples and arguments present instances of externalization, that is, when external processes are created or utilized to perform some function for the organism in question. Many such examples are instances of niche construction. As Laubichler and Renn (2015) point out, the reverse is equally imported, where external processes are internalized. For instance, the mitochondria in eukaryotic cells derive from biological entities, essentially bacteria that were originally independent, but then incorporated into those eukaryotic cells for metabolic processes. More generally, regulatory networks become ever more sophisticated to control ever more complex internal processes.
8. In the same direction, the answer to the question why the simulation of protein folding is so difficult is probably not physical (an energy landscape with many metastable states), but genuinely biological: The energy landscape evolved to provide flexibility to switch between different conformations.
9. Biological processes can control and regulate each other not only hierarchically, but also reciprocally. Processes and constraints can switch their roles, both between and within time scales (Montévil and Mossio 2015). That is, what is controlled and regulated, and what is controlling may depend on the perspective. Such reciprocity is a fundamental aspect of biological life, within cells and organisms up to the scale of the biosphere.

Thus, we see that when viewed from the perspective of the above theses, many very diverse biological phenomena fall in place conceptually and acquire evolutionary significance.

## Evolution

From the perspective of biological evolution, the most important process is reproduction. The basic growth law for reproduction is given by the exponential function. When the exponent is positive, the lineage or whatever is reproducing is expanding, and who has the highest exponent expands fastest and wins out over the others. But in order to expand, the process needs to draw matter and energy from outside. Since those are limited, this inevitably causes competition for scarce resources.

By the Darwinian paradigm (Darwin 1985), competition causes selection. Some species succeed and expand. While a stone, for instance, cannot expand, a biological species (or a bacterial colony or a virus population) can, because its members can control processes outside themselves. The reproduction of the control requires the transmission of information. In contrast to matter or energy, information does not obey a conservation law, and therefore, one organism may produce many offspring. Biological information transmission (a topic analyzed in detail in (Jost 2020) both requires and ensures a certain regularity or repeatability, because the transmitted information cannot be modified too much without becoming useless, unless by chance it hits upon a new control. Again, the modifiability can in principle be controlled itself, which leads to the issue of evolvability, which, however, will be pursued elsewhere. Here, we only point out that as a consequence of this regularity and repeatability, biological processes are typically periodic at some scale, perhaps with small variations.
